# The Roles of Histone Demethylase Jmjd3 in Osteoblast Differentiation and Apoptosis

**DOI:** 10.3390/jcm6030024

**Published:** 2017-02-23

**Authors:** Di Yang, Bo Yu, Haiyan Sun, Lihong Qiu

**Affiliations:** Department of Endodontics, School of Stomatology, China Medical University, Shenyang 110002, China; dd173433281@163.com (B.Y.); m13066784163@163.com (H.S.); drqlh@yahoo.com (L.Q.)

**Keywords:** Jmjd3, H3K27me3, osteoblast differentiation, bone formation, osteoblast apoptosis

## Abstract

Posttranslational modifications including histone methylation regulate gene transcription through directly affecting the structure of chromatin. Trimethylation of histone 3 lysine 27 (H3K27me3) is observed at the promoters of a wide variety of important genes, especially for mammalian development, and contributes to gene silencing. Demethylase Jumonji domain-containing 3 (Jmjd3) catalyzes the transition of H3K27me3 to H3K27me1, therefore from a repressive to an active status of gene expression. Jmjd3 plays important roles in cell differentiation, inflammation, and tumorigenesis by targeting distinct transcription factors. In this review, we summarize the pivotal roles of Jmjd3 in maintaining skeletal homeostasis through regulating osteoblast differentiation, maturation, and apoptosis.

## 1. Introduction

Eukaryotic DNA is packed into chromatin, the basic repeating unit of which is the nucleosome. Each nucleosome core particle consists of eight histone proteins—two molecules each of histones H2A, H2B, H3, and H4—and double-stranded DNA that is 146 nucleotide pairs long [1]. All four of the histones (H2A, H2B, H3, and H4) are relatively small proteins (102–135 amino acids). They share a structural motif, known as the histone fold [2].

In addition to the histone fold, each of the core histones has a long N-terminal amino acid “tail”, which extends out from the DNA-histone core [3]. These histone tails are subjected to several different types of modifications including methylation, acethylation, phosphorylation, and ubiquitination, which control many aspects of the chromatin structure [4,5]. These nucleosome modifications are added and removed by enzymes; for example, methyl groups are added to the histone tails by histone methyl transferase (HMTs) and taken off by histone demethylase (HDMs) [6]. The various modifications of the histone tails have several important consequences. The most profound effect of modified histone tails is their ability to attract specific proteins to a stretch of chromatin that has been appropriately modified.

Histone methylation occurs on lysines and arginines. Residues can exist in different methylated forms with lysines (K), being either mono- (me1), di- (me2), or tri-methylated (me3), and arginines (R) being mono-methylated or symmetrically or asymmetrically di-methylated [7]. In general, trimethylation of H3K4, H3K36, and H3K79 is found in euchromatic regions with transcriptional activity, whereas H3K9me3/me2, H4K20me3, and H3K27me3 are associated with transcriptionally silenced chromatin [7,8]. Histone modifications are believed to be important for coordinating both transient changes in gene transcription, as well as for maintaining differential patterns of gene expression during organismal development. Jumonji domain-containing 3 (Jmjd3) has been identified as a histone demethylase, which specifically catalyzes the removal of methylation from H3K27me3 [9,10]. Although Jmjd3 was shown to play important roles in various cellular events including in the immune system and cancer [5,7], the roles of Jmjd3 in osteoblasts have not been described. This review will primarily focus on the function of Jmjd3 in osteoblast differentiation and apoptosis.

## 2. Jmjd3 and Osteogenic Differentiation of Mesenchymal/Stromal Stem Cells (MSCs)

The skeleton undergoes continuous bone remodeling involving the destruction of mineralized bone followed by the formation and mineralization of the new bone matrix [11]. The homeostasis of bone remodeling depends on the mesenchymal osteoblasts and hematopoietic osteoclasts, which are strictly regulated by a complex signaling network. In order to keep both processes in balance, the differentiation and apoptosis in both cell types are tightly regulated with the profound changes of gene expression [12,13].

Jmjd3 is regulated by a variety of differentiation cues and stress signals during bone remodeling, including bone marrow MSC osteogenic differentiation [14], osteoblast differentiation [15,16], bone formation [15,16,17], and osteoblast apoptosis [18]. MSCs are multipotent progenitor cells with self-renewal ability and multilineage differentiation potential, including osteogenesis, chondrogenesis, and adipogenesis. The commitment of MSCs to an osteogenic lineage requires the coordinated inhibition of differentiation to the adipogenic lineage. Gene expression profiling revealed that HOX and distal-less homeobox 5 (DLX5) have essential roles in MSC osteogenic differentiation which is regulated by Jmjd3 through the removal of H3K27me3 on their promoter regions. During MSC osteogenic differentiation, Jmjd3 expression is induced by bone morphogenic protein 4 and 7 (BMP4/7), which are potent inducers of osteogenic differentiation of MSCs. Furthermore, Jmjd3 directly regulates BMP2/4 expression by removing H3K27me3 marks [14]. These results suggest a possible feedback system in Jmjd3-regulated MSC fate commitment to osteogenic cells.

## 3. Jmjd3 and Osteoblast Differentiation

**Figure 1:**
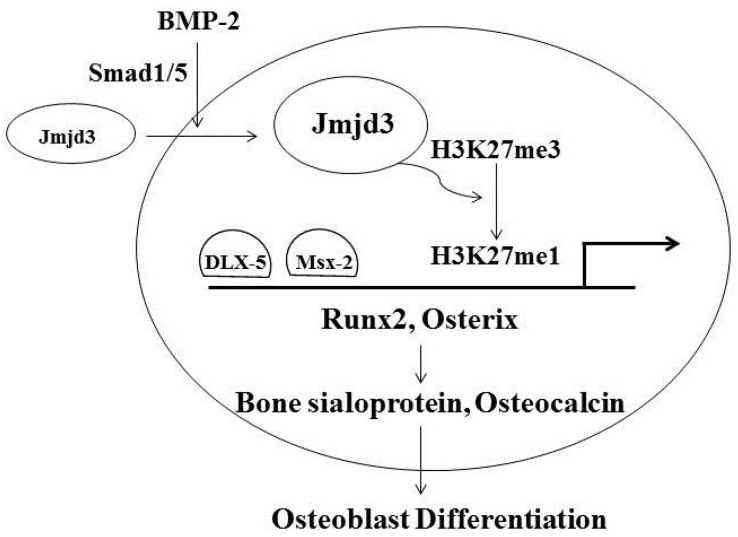
Jmjd3 regulates osteoblast differentiation through transcription factors Runx2 and Osterix. BMP-2 induces Jmjd3 expression and translocation into the nucleus, where the level of H3K27me3 on the promoter regions of Runx2 and Osterix decreases by demethylation. Thus, this further promotes Msx-2 and Dlx-5 to approach to the binding sites of these promoters, resulting in the up-regulations of Runx2 and Osterix.

Both Runx2 and Osterix are important transcription factors for osteoblast differentiation and control the expressions of bone-related genes such as Bone sialoprotein (BSP) and Osteocalcin (OCN). These bone-related genes are required for terminal osteoblast differentiation and bone mineralization [19,20,21]. In an in vitro study, Jmjd3 expression and nuclear localization were stimulated during osteoblast differentiation. Noggin, which is an extracellular antagonist of BMP-2, inhibited Jmjd3 expression in the cells cultured in the osteoblast differentiation medium [15]. Upon activation of the BMP pathway, Smad1 and Smad5 are phosphorylated and interact with Smad4 to enter the nucleus to regulate their target genes [22]. Silencing of Smad1/5 decreased Jmjd3 expression, which suggests that BMP-2 signaling is involved in the induction of the Jmjd3 expression during osteoblast differentiation. Silencing of Jmjd3 expression suppressed osteoblast differentiation through increasing the occupation of H3K27me3 to the promoter regions of Runx2 and Osterix [15]. On the promoter regions of Runx2 and Osterix, there are several important binding sites for transcription factors such as Msx-2 and Dlx-5. The increased level of repressive transcription mark H3K27me3 might further prevent Msx-2 and Dlx-5 from approaching to these binding sites, resulting in the down-regulation of Runx2 and Osterix. Introduction of exogenous Runx2 and Osterix partly recovered the expressions of BSP and OCN and, sequentially, osteoblast differentiation in the Jmjd3 knockdown cells [15]. These results provide new findings that Jmjd3 plays important roles in osteoblast differentiation via transcription factors Runx2 and Osterix (Figure 1).

## 4. Jmjd3 and Bone Formation

Osteoblast maturation is essential for intramembranous and endochondral bone formation. Administration of small interfering RNA targeting Jmjd3 into the mouse calvarial region decreased the bone mineral density, bone mineralization rate, bone thickness, and mineralized bone matrix [15]. Jmjd3 expression primarily in the nuclei of osteoblasts increased from E14.5 to E18.5 and the parietal bones of Jmjd3^−/−^ mouse embryos exhibited less mineralization. Jmjd3^−/−^ mice exhibited open fontanelles, less mineralized cranial bones and hypoplastic clavicles at E18.5. Biochemical and genetic methods demonstrated that Jmjd3 mediated Runx2 transcriptional activity and cooperated with Runx2 to promote osteoblast differentiation and bone formation in vivo [16]. The differentiation of osteoclasts, which are terminally differentiated cells that are responsible primarily for bone resorption, constitutes the other important part of skeletal homeostasis. Yasui et al. revealed that demethylation of H3K27me3 in the *Nfatc1* gene locus by Jmjd3 plays a critical role in RANKL-induced osteoclast differentiation [17]. Further understanding of the mechanism of both osteoblast and osteoclast differentiation regulated by Jmjd3 will be extremely useful to uncover the mechanism of bone homeostasis.

## 5. Jmjd3 and Osteoblast Apoptosis

Loss of growth factors and cytokines activates cell intrinsic apoptotic pathway by disrupting the integrity of the mitochondrial membrane. The integrity of the mitochondrial membrane is controlled by the B cell lymphoma-2 (Bcl-2) family of proteins including anti-apoptotic (Bcl-2, Bcl-xL, etc.) and pro-apoptotic members. The latter are divided into two subgroups as follows: the multi-domain members (Bax, Bak, etc.) and the Bcl-2 homology 3 domain-only members such as Bim [23]. Bim is essential for the initiation of apoptosis on the upstream of Bax and Bak activation [24]. Bim expression is regulated by both transcriptional and posttranslational mechanisms to prevent inappropriate apoptosis. Phosphorylation of Bim at Ser65 by extracellular regulated protein kinases 1/2 (ERK1/2) reduces the pro-apoptotic potency of Bim, whereas phosphorylation of Bim at Thr112 by c-Jun *N*-terminal kinase (JNK) induces Bim pro-apoptotic activity [25]. Bcl-2 was down-regulated in the Jmjd3 knockdown cells with an increased level of H3K27me3 on its promoter regions. Knockdown of Jmjd3 did not affect the expression of Bim at the transcriptional level; however, the phosphorylation of Bim was down-regulated in Jmjd3 knockdown cells as well as the phosphorylation of ERK1/2. Moreover, Protein kinase D1 (PKD1) was revealed to link between histone demethylase Jmjd3 and the ERK1/2 signaling pathway to phosphorylate Bim and protect from osteoblast apoptosis [18]. This study highlights the pivotal roles of Jmjd3 in regulating osteoblast apoptosis through targeting Bcl-2 expression and Bim phosphorylation.

Collectively, in addition to the important roles of Jmjd3 in cell differentiation [7,26,27], inflammation [28,29], and tumorigenesis [5,30], the current studies introduced in this review disclosed the pivotal roles of Jmjd3 in maintaining skeletal homeostasis through regulating osteoblast differentiation, maturation, and apoptosis. Further studies are important to determine how Jmjd3 orchestrates bone formation and metabolism under both physiological and pathological conditions.
